# Socioeconomic Deprivation’s Effect on Depression: Family Satisfaction’s Moderating Role

**DOI:** 10.3390/bs16091463

**Published:** 2026-08-24

**Authors:** Sung-Su Han, Sung-Man Bae

**Affiliations:** 1 Department of Psychology, Graduate School, Dankook University, Cheonan 31116, Republic of Korea; radery62@gmail.com; 2 Department of Psychology, College of Health Science, Dankook University, Cheonan 31116, Republic of Korea

**Keywords:** socioeconomic deprivation, depression, family relationship satisfaction, Basic Livelihood Security System, longitudinal study

## Abstract

Socioeconomic deprivation has been associated with depression, and previous studies have demonstrated an association between family relationship satisfaction and depression. However, there is a lack of consensus regarding whether family relationship satisfaction can moderate the association between socioeconomic deprivation and depression. This study examined the longitudinal association between socioeconomic deprivation and depression and tested whether family relationship satisfaction and benefits from the Basic Livelihood Security System moderated this association. Data from the Korea Welfare Panel Study were used, including 7867 Korean adults followed over a five-year period from 2017 to 2021. Correlation analysis and generalized estimating equations were conducted to examine whether family relationship satisfaction and Basic Livelihood Security System benefits moderated the association between socioeconomic deprivation and depression. Socioeconomic deprivation was significantly associated with higher depressive symptoms, whereas family relationship satisfaction was significantly associated with lower depressive symptoms. The interaction between socioeconomic deprivation and family relationship satisfaction was significant (B = −0.174, Wald χ^2^ = 37.662, *p* < 0.001), indicating that family relationship satisfaction moderated this association. Although receipt of Basic Livelihood Security System benefits was significantly associated with depression, the interaction between socioeconomic deprivation and benefit receipt was not significant (B = 0.135, Wald χ^2^ = 2.506, *p* = 0.113). These findings highlight the importance of considering both socioeconomic conditions and family relationship resources when examining factors associated with depressive symptoms among socioeconomically disadvantaged adults.

## 1. Introduction

According to an announcement by the National Health Insurance Service in 2023 on the Status of Depression Treatment, the number of patients with major depressive disorder has increased by 32.9% in the past five years, reaching a record high of 1,007,744 individuals in Korea. This is the first time that the number of patients with depressive disorders has exceeded one million (National Health Insurance Service, 2023). In particular, the number of adults who reported depressive symptoms increased by 31.4% over the same period (National Health Insurance Service, 2023). Depressive symptoms contribute to the formation of negative self-concepts and can induce insomnia and a sense of helplessness, thereby adversely affecting mental health (Reed-Fitzke, 2020; Sivertsen et al., 2012; Ozment & Lester, 2001). They also lead to decreased job productivity and increased risk of unemployment (Lépine & Briley, 2011). Moreover, if depressive symptoms persist and worsen, they can trigger suicidal thoughts and attempts (Orsolini et al., 2020).

According to integrated models, such as the biopsychosocial model and stress vulnerability model, depression arises from the interaction of environmental factors such as negative experiences in childhood and workplace conditions (Giano et al., 2021; Siegrist & Wege, 2020), personal factors such as stress and loneliness (Black et al., 2015; Erzen & Çikrikci, 2018), and physiological factors such as inflammation and serotonin depletion (Moncrieff et al., 2023; Slavich & Irwin, 2014). Socioeconomic deprivation is a significant environmental factor associated with depression (Ye et al., 2021). It is defined as a state in which individuals’ needs across various domains remain unfulfilled because of a lack of material and social resources. This expanded concept of deprivation moves beyond material factors and acknowledges the influence of both material and sociocultural factors (Townsend, 1987). Individuals experiencing socioeconomic deprivation often encounter a lack of resources in various areas, such as finance, healthcare, social security services, and interpersonal relationships, leading to susceptibility to feelings of helplessness and depression (Fernández-Niño et al., 2014; J. H. Kim et al., 2015; Ostler et al., 2001).

While the association between economic deprivation and depression has been well established (Lorant et al., 2007), there is insufficient evidence to directly link socioeconomic deprivation to depression (Ye et al., 2021). Studies focusing on older adults have reported an association between socioeconomic deprivation and depression (K. Kim, 2021; Son, 2018). Furthermore, S. B. Lee et al. (2021) found a close association between depression in young adults and socioeconomic deprivation, encompassing housing, occupation, and economic aspects. Taken together, previous studies have documented associations between socioeconomic deprivation and depression across different age groups. However, less is known about the factors that may modify this association, particularly family relationship resources and public income support.

South Korea has made efforts to alleviate the problems caused by environmental factors through the Basic Livelihood Security System (Ministry of Government Legislation, 2023). Studies have suggested that a Basic Livelihood Security System is associated with reductions in the economic challenges faced by vulnerable populations experiencing deprivation and may be associated with better mental health through income support (Nam & Lee, 2020). However, entering the Basic Livelihood Security System was found to be a source of economic stress owing to deprivation, leading to increased stress and depressive symptoms (W. Lee, 2010). Thus, the role of the Basic Livelihood Security System in the relationship between socioeconomic deprivation and depression remains unclear.

Previous research has suggested that socioeconomic deprivation may adversely affect family relationships, which in turn may be associated with depression, supporting a potential mediating pathway (K. Kim, 2021). However, this pathway does not preclude the possibility that family relationship satisfaction also functions as a resource that alters the strength of the association between socioeconomic deprivation and depression. From the perspective of the ABC-X model, family resources can influence how individuals respond to socioeconomic stressors. Accordingly, the present study focused on a moderating rather than mediating role of family relationship satisfaction to examine whether the association between socioeconomic deprivation and depression differs according to the level of family relationship satisfaction.

Accordingly, it is important to examine factors that may modify the association between socioeconomic deprivation and depression in adults. Positive family relationships have been identified as a significant factor associated with lower levels of depression, potentially by providing emotional stability (Cardoso-Moreno & Tomás-Aragones, 2017). However, the ABC-X model (McCubbin & Patterson, 2014) suggests that even in similar stressful situations (A), differences in actual family resources (B) and perceived family resources (C) can lead to varying reactions to actual stress (X). Building on prior research emphasizing the close relationship between perceived social support and lower levels of depression (Santini et al., 2015), it is necessary to examine how subjective satisfaction with family relationships is associated with depression. Despite economic difficulties, higher satisfaction with family relationships was associated with lower levels of depression (Park, 2022). Moreover, higher subjective satisfaction with one’s spouse was associated with a more pronounced alleviating effect on depression (D. Kim & Ki, 2019). However, it was also found that negative external events experienced by families, such as socioeconomic deprivation, may be associated with poorer family relationships and higher levels of depression (K. Kim, 2021). Despite evidence supporting the potential of family relationship satisfaction to be associated with lower depression, its role in the association between socioeconomic deprivation and depression remains unclear.

To clearly understand the moderating roles of assistance from the Basic Livelihood Security System and satisfaction with family relationships in the association between socioeconomic deprivation and depression, it is essential to consider control and demographic variables. Previous research has shown a tendency for increased depression with increasing age (Stordal et al., 2003), and a higher prevalence of depression in women than in men (Anguzu et al., 2021). Among internal factors, self-esteem was identified as a prominent protective factor against depression (Orth & Robins, 2022); high self-esteem reduced depression through the formation of a positive self-concept (Kernis et al., 1991).

Although previous studies have documented the association between socioeconomic deprivation and depression across different populations, relatively little is known about whether family relationship satisfaction and public income support modify this association over time. In particular, evidence examining these potential moderators using repeated observations across multiple time points remains limited. This study aimed to examine longitudinally whether family relationship satisfaction and the Basic Livelihood Security System moderate the association between socioeconomic deprivation and depression.

**H1.**  *Socioeconomic deprivation will be positively associated with depression.*

**H2.**  *Family relationship satisfaction will moderate the association between socioeconomic deprivation and depression, such that the positive association between socioeconomic deprivation and depression will be weaker at higher levels of family relationship satisfaction.*

**H3.**  *Receipt of Basic Livelihood Security System benefits will moderate the association between socioeconomic deprivation and depression, such that the positive association between socioeconomic deprivation and depression will be weaker among recipients than among non-recipients.*

## 2. Materials and Methods

This study used data from the 12th (2017), 14th (2019), and 16th (2021) waves of the Korea Welfare Panel Study (KoWePS), conducted by the Korea Institute for Health and Social Affairs and the Institute of Social Welfare at Seoul National University. These three waves were selected at two-year intervals to examine the study variables longitudinally while maintaining a consistent time interval between observations. The KoWePS was established using 7072 households selected through stratified sampling based on the 2006 National Living Conditions Survey, with low-income households oversampled to ensure adequate representation. Surveys were conducted annually from February to May using face-to-face interviews, supplemented by telephone and proxy interviews when necessary. A total of 12,938 adults participated in the 12th wave (2017). To construct a balanced longitudinal panel, participants were required to have participated in all three survey waves (2017, 2019, and 2021). In addition, participants with missing family relationship satisfaction data at any of the three waves were excluded because family relationship satisfaction was a key study variable. Consequently, the final analytic sample consisted of 7867 adults. The analytic sample included 3127 men (39.7%) and 4740 women (60.3%). At baseline (2017), the mean age was 60.25 years (SD = 15.17). Missing data were not imputed. For variables other than family relationship satisfaction, observations with missing values required for a given analysis were excluded from that analysis. Although this study used publicly available secondary data, ethical approval was obtained from the Institutional Review Board of Dankook University (Approval No. DKU 2024-12-032-002).

This study used a measure of socioeconomic deprivation that was adapted based on K. Kim’s (2021) study and modified from Heo et al.’s (2010) study. Constructed with items corresponding to Townsend’s delineation of deprivation across various domains, socioeconomic deprivation was assessed using 21 items across six domains: nutrition (5 items), housing (7 items), social security (2 items), occupational and economic (3 items), health and medical (2 items), and social deprivation (2 items). Each item was scored as 1 if deprivation was experienced and 0 otherwise, and the item scores were summed across the six domains to calculate the total socioeconomic deprivation score. Consistent with the scoring approach used in the adapted measure, no differential weights were assigned to individual items or domains. The item scores were summed to yield a total score ranging from 0 to 21. A higher total score indicated a higher level of socioeconomic deprivation. The full set of items and their coding criteria are provided in Supplementary Table S1.

Family relationship satisfaction comprised four domains: family life, spouse, children, and children’s sibling relationships. Example items assessed satisfaction with family life and satisfaction with the relationship with one’s spouse. Each item was rated on a scale from 1 (very dissatisfied) to 7 (very satisfied). Because the applicability of the items varied according to participants’ family structure, items that were not applicable were treated as missing. Family relationship satisfaction was calculated as the mean of the applicable items, resulting in a score ranging from 1 to 7 regardless of the number of applicable items. A higher score indicated a higher level of subjective satisfaction with family relationships. The overall reliability coefficients for family relationship satisfaction, measured using Cronbach’s alpha, were 0.841 for the 12th wave, 0.835 for the 14th wave, and 0.837 for the 16th wave.

The receipt of benefits from the Basic Livelihood Security System was assessed by considering the characteristics of the Korean Welfare Panel data presented by Choi (2018). Data from the previous year for each wave were used to confirm eligibility for the Basic Livelihood Security System benefits. In other words, to determine the status of the Basic Livelihood Security System benefits in the 12th wave, the values provided in the 11th wave were applied. Receipt of benefits in each domain was integrated, and a score of 1 was assigned if benefits were received in that year and 0 otherwise.

Depression was assessed using the CES-D-11 (Center for Epidemiologic Studies Depression Scale), which consists of 11 self-report questions regarding an individual’s psychological state over the past week. Example items include “I felt depressed” and “I felt sad.” The response categories are 0 points (less than 1 day per week), 1 point (1–2 days per week), 2 points (3–4 days per week), and 3 points (5 days or more per week). Two items (I felt I was doing well and I enjoyed life) were reverse-coded, with higher scores indicating a higher level of depression. The overall reliability coefficients (Cronbach’s alpha) for the CES-D-11 were 0.874 for the 12th wave, 0.872 for the 14th wave, and 0.880 for the 16th wave.

Self-esteem was measured using the Rosenberg Self-Esteem Scale. Example items include “I feel that I am a person of worth, at least on an equal plane with others” and “I feel that I have a number of good qualities.” This scale assesses respondents’ psychological state at the time of the survey, with response categories of 1 point (strongly disagree), 2 points (disagree), 3 points (agree), and 4 points (strongly agree). Five of the 10 items are reverse-coded, such that higher total scores indicate higher levels of self-esteem. The overall reliability coefficients (Cronbach’s alpha) for the scale were 0.744 for the 12th wave, 0.751 for the 14th wave, and 0.770 for the 16th wave.

IBM SPSS Statistics 27 was used for analysis; descriptive statistics were used to analyze demographic characteristics of the measured variables, and Cronbach’s alpha was calculated to assess the reliability of the measurement scales. Correlation analyses were conducted to examine associations among the study variables. Generalized estimating equations (GEEs) were used to account for repeated observations nested within participants across the three survey waves and to examine whether family relationship satisfaction and Basic Livelihood Security System benefits moderated the association between socioeconomic deprivation and depression. Each participant was specified as a cluster, with observations from 2017, 2019, and 2021 entered as repeated measurements within participants. Age, gender, and self-esteem were included as covariates, and continuous predictor variables were entered in their original metric without mean-centering prior to constructing the interaction terms. An independent working correlation structure was selected based on the lowest quasi-likelihood under the independence model criterion (QIC) among the candidate structures presented in Table 1. Although the independent working structure assumes no within-participant correlation in the working correlation matrix, robust covariance estimation was used to account for potential misspecification of the working correlation structure and to obtain robust standard errors (Pan, 2001; Pak & Bae, 2023).

## 3. Results

### 3.1. Sociodemographic Characteristics

To examine potential selection bias, baseline characteristics were compared between participants included in the final analytic sample (n = 7867) and those excluded (n = 5071). As shown in Table 2, significant differences were observed between the two groups in age, gender, socioeconomic deprivation, depressive symptoms, family relationship satisfaction, self-esteem, and receipt of Basic Livelihood Security System benefits. Compared with the included participants, excluded participants were younger and had slightly higher levels of socioeconomic deprivation and depressive symptoms. They also had lower family relationship satisfaction and self-esteem and were more likely to receive Basic Livelihood Security System benefits. In addition, the excluded group had a higher proportion of males.

Table 3 presents the demographic characteristics and key study variables of the final analytic sample across the three survey waves. The same 7867 participants were included in all three waves. At baseline in 2017, the mean age was 60.25 years (SD = 15.17), and 3127 participants (39.7%) were male and 4740 (60.3%) were female. In addition, 505 participants (6.4%) received Basic Livelihood Security System benefits, whereas 7362 (93.6%) did not. Socioeconomic deprivation scores were positively skewed across all three waves. The observed score ranges were 0–12 in 2017 (M = 1.33, SD = 1.24), 0–14 in 2019 (M = 1.29, SD = 1.21), and 0–11 in 2021 (M = 1.25, SD = 1.19), with skewness values of 1.86, 2.18, and 1.53, respectively.

### 3.2. Correlation Analysis 

Table 4 presents the means, standard deviations, and correlations among the key variables for the three survey waves. Across all three waves, depression was positively correlated with socioeconomic deprivation (r = 0.289 in 2017, r = 0.260 in 2019, and r = 0.273 in 2021, all *p* < 0.001) and negatively correlated with family relationship satisfaction (r = −0.407, r = −0.340, and r = −0.353, respectively, all *p* < 0.001). Receipt of Basic Livelihood Security System benefits was also positively correlated with socioeconomic deprivation across all three waves (r = 0.289, r = 0.305, and r = 0.288, respectively, all *p* < 0.001).

### 3.3. Moderation Analysis

To examine the moderating effects of family relationship satisfaction and Basic Livelihood Security System benefits moderated the association between socioeconomic deprivation and depression, GEEs were used in SPSS 27.0. The results are presented in Table 5 and Table 6. Socioeconomic deprivation was significantly and positively associated with depression (B = 0.485, 95% CI [0.432, 0.539], Wald χ^2^ = 317.613, *p* < 0.001), whereas family relationship satisfaction was significantly and negatively associated with depression (B = −0.685, 95% CI [−0.764, −0.607], Wald χ^2^ = 292.489, *p* < 0.001). Moreover, the interaction between socioeconomic deprivation and family relationship satisfaction was statistically significant (B = −0.174, 95% CI [−0.229, −0.118], Wald χ^2^ = 37.662, *p* < 0.001). This finding indicates that the association between socioeconomic deprivation and depression varied according to the level of family relationship satisfaction.

The results of the analysis of the moderating role of Basic Livelihood Security System benefits in the association between socioeconomic deprivation and depression are presented in Table 7 and Table 8. Socioeconomic deprivation was significantly positively associated with depression (B = 0.471, Wald χ^2^ = 287.102, *p* < 0.001), while receipt of Basic Livelihood Security System benefits was significantly positively associated with depression (B = 1.281, Wald χ^2^ = 62.356, *p* < 0.001). However, the interaction between socioeconomic deprivation and Basic Livelihood Security System benefits was not statistically significant (B = 0.135, Wald χ^2^ = 2.506, *p* = 0.113).

## 4. Discussion

This study aimed to examine whether of family relationship satisfaction and Basic Livelihood Security System benefits on association between socioeconomic deprivation and depression.

Higher socioeconomic deprivation was significantly associated with increased depressive symptoms over time. This aligns with previous research reporting an association between socioeconomic deprivation and depression (Jani et al., 2012; Ye et al., 2021). Regardless of actual income, severe socioeconomic deprivation reduces the resources available for the fulfillment of needs (Holmgren et al., 2021; Ye et al., 2021). These results highlight the importance of considering socioeconomic deprivation and access to necessary resources when addressing depressive symptoms among adults.

Second, family relationship satisfaction was significantly negatively associated with depression. This is consistent with previous findings showing an association between higher family relationship satisfaction and lower levels of depression (Røsand et al., 2012; Weitlauf et al., 2014). Positive perceptions of family relationships, regardless of the actual support received, may serve as an internal resource for maintaining mental health. Such positive perceptions may also be associated with greater self-efficacy in coping with stress (Yoo & Sung, 2009).

Third, the interaction between socioeconomic deprivation and family relationship was statistically significant, indicating a moderating association. This indicates that the association between socioeconomic deprivation and depression varies depending on satisfaction with family relationships. Higher satisfaction with family relationships as a social resource may be associated with greater individual self-efficacy. Consequently, even under adverse socioeconomic conditions, perceived social support, including satisfaction with family relationships, may be associated with more adaptive coping with daily stress and better mental health (Santini et al., 2015). In terms of the magnitude of the moderating association, the interaction coefficient (B = −0.174) indicates that each one-point increase in family relationship satisfaction was associated with a 0.174-point decrease in the slope relating socioeconomic deprivation to CES-D-11 scores. Although this coefficient provides information about the magnitude of the observed moderating association, its clinical significance cannot be established from the present analyses alone and should therefore be interpreted cautiously.

Fourth, receipt of benefits from the Basic Livelihood Security System was significantly positively associated with depression. This positive association should not be interpreted as evidence that Basic Livelihood Security System benefits increase depressive symptoms, as benefit recipients may differ systematically from non-recipients in socioeconomic and other characteristics associated with depression. However, the benefits did not moderate the association between socioeconomic deprivation and depression, indicating that the present study did not find evidence that Basic Livelihood Security System benefit receipt buffered the association between socioeconomic deprivation and depressive symptoms. The findings regarding the Basic Livelihood Security System should also be interpreted with caution. Receipt of Basic Livelihood Security System benefits is not randomly assigned but is determined by eligibility criteria related to socioeconomic circumstances. Therefore, recipients may systematically differ from non-recipients in characteristics such as the severity of economic hardship, health status, and family circumstances. Although the present analyses adjusted for several covariates, they were not designed to eliminate selection bias between recipients and non-recipients. Accordingly, the observed association between Basic Livelihood Security System receipt and depression should not be interpreted as evidence of a direct causal effect of the benefits on depression.

Given the relatively large sample size, statistical significance should be distinguished from clinical or practical significance. Although the observed associations and moderating effect were statistically significant, their clinical significance cannot be determined from the present analyses alone. Therefore, the magnitude and practical importance of these associations should be interpreted cautiously, and further research is needed to determine their clinical relevance.

This study made several important contributions and its results have important implications. First, it demonstrated a longitudinal association between socioeconomic deprivation and depression in an adult sample spanning a broad age range. Previous studies have often examined socioeconomic deprivation in specific groups such as middle-aged individuals or older adults and individuals living alone. Consequently, these studies provide limited evidence regarding socioeconomic deprivation in the broader adult population (Im & Park, 2018; S. B. Kim & Park, 2021; S. Y. Kim et al., 2018). This study controlled for demographic variables and self-esteem by analyzing longitudinal data over a 5-year period. The association between socioeconomic deprivation and depression remained significant after adjustment for demographic variables and self-esteem, supporting the relevance of socioeconomic deprivation to depressive symptoms in adulthood.

Second, this study identified a significant longitudinal moderating role of family relationship satisfaction in the association between socioeconomic deprivation and depression. This finding suggests that family relationship satisfaction may represent an important social resource in understanding differences in depressive symptoms among adults experiencing socioeconomic deprivation. However, the present findings do not establish that interventions aimed at increasing family relationship satisfaction would reduce depression. Family relationship satisfaction may reflect not only individuals’ subjective perceptions but also actual family support, relationship quality, household stability, and shared economic resources. Therefore, practical approaches should consider broader family and social resources, including family-based support, access to social services, economic security, and appropriate support for individuals with limited family resources, including those living alone.

### Limitations and Future Directions

Several limitations of this study should be considered. First, socioeconomic deprivation was measured by dichotomously coding each deprivation experience as present or absent. Although this approach captures the cumulative number of deprivation experiences, it does not account for differences in the severity or degree of difficulty associated with each experience. Consequently, individuals experiencing different levels of severity may receive the same score for a given deprivation item. Future research should consider measures that capture the degree or severity of deprivation, such as ordinal response scales, to provide a more nuanced assessment of socioeconomic deprivation. The present findings should also be revalidated using alternative or standardized measures of socioeconomic deprivation.

Second, receipt of Basic Livelihood Security System benefits was measured as a binary indicator (recipient vs. non-recipient), without considering the amount or value of benefits received. Therefore, this measure may not fully capture differences in the level of economic support among recipients. Future research should consider more detailed measures of benefit receipt to better examine its association with depression.

Third, this study did not simultaneously consider both actual family resources and the subjective evaluation of family resources. Therefore, further research is necessary to examine both adults’ subjective satisfaction with family relationships and the actual resources provided by the family, as well as how these factors may jointly relate to socioeconomic deprivation and depression.

Fourth, although this study employed longitudinal data, the possibility of reverse causality cannot be completely excluded. Depression may not only be associated with socioeconomic deprivation but may also contribute to worsening socioeconomic conditions through reduced occupational functioning, social participation, and income, and may influence individuals’ perceptions of deprivation. Similarly, depressive symptoms may negatively influence individuals’ perceptions of family relationships, leading to lower reported family relationship satisfaction. Future studies using cross-lagged panel models or other longitudinal approaches that better examine temporal ordering are needed to clarify the bidirectional relationships among socioeconomic deprivation, family relationship satisfaction, and depression.

Fifth, the generalizability of the findings regarding the Basic Livelihood Security System may be limited beyond the South Korean context. The Basic Livelihood Security System operates within a specific national welfare system with particular eligibility criteria and benefit structures. Therefore, the associations observed in this study may not be directly generalizable to countries with different welfare systems or public assistance programs. Future cross-national research is needed to examine whether similar patterns are observed under different welfare policy contexts.

## 5. Conclusions

This study examined the longitudinal association between socioeconomic deprivation and depression using data from the Korea Welfare Panel Study. Socioeconomic deprivation was significantly associated with depression over time, and family relationship satisfaction moderated this association, such that the positive association between socioeconomic deprivation and depression was weaker at higher levels of family relationship satisfaction. Although receipt of Basic Livelihood Security System benefits was significantly associated with depression, it did not moderate the association between socioeconomic deprivation and depression. These findings highlight the importance of considering both socioeconomic conditions and family relationship satisfaction when examining depressive symptoms among adults. The findings also suggest the relevance of considering broader family and social resources, including relationship quality, family support, access to social services, and economic security, rather than focusing solely on individuals’ perceptions of their families. Future research should further clarify the bidirectional relationships among these variables using longitudinal designs that explicitly model reciprocal effects.

## Figures and Tables

**Table 1:** QIC for working correlation matrix selection.

Correlation	QIC
Independent	330,449.131
Autoregressive	330,726.844
Unstructured	330,855.352
Exchangeable	330,863.774

**Table 2:** Baseline characteristics of participants included in and excluded from the final analytic sample.

Baseline Characteristic	Included (n = 7867)	Excluded (n = 5071)	Test Statistic	*p*
Age	60.25 (15.17)	48.93	*t* = 31.25	0.000
Gender			χ^2^ = 156.00	0.000
Male	3127 (39.7)	2582 (50.9)		
Female	4740 (60.3)	2489 (49.1)		
Socioeconomic Deprivation	1.33 (1.24)	1.38 (1.43)	*t* = −2.00	0.046
CES-D-11	3.20 (4.38)	3.80 (5.14)	*t* = −6.37	0.000
Family relationship satisfaction	5.47 (0.92)	5.25 (1.09)	*t* = 8.34	0.000
Self-esteem	30.64 (3.79)	30.08 (4.43)	*t* = 6.87	0.000
Basic Livelihood Security System			χ^2^ = 149.47	0.000
Non-recipient	7362 (93.6)	4428 (87.3)		
Recipient	505 (6.4)	643 (12.7)		

Note. Values are presented as mean (SD) or n (%), as appropriate. Welch’s *t*-tests were used for continuous variables, and Pearson’s chi-square tests were used for categorical variables. Sample sizes varied due to missing baseline data (CES-D-11 and self-esteem: included n = 7867, excluded n = 4161; family relationship satisfaction: included n = 7867, excluded n = 2076). CES-D-11 = 11-item Center for Epidemiologic Studies Depression Scale.

**Table 3:** Characteristics of the analytic sample across the three survey waves (n = 7867).

Variables	2017 Year		2019 Year		2021 Year	
Gender						
Male	3127				39.7%	
Female	4740				60.3%	
Age, mean (SD)	60.25 (15.17)		62.25 (15.17)		64.25 (15.17)	
Basic Livelihood Security System						
Yes	505	6.4%	554	7.0%	601	7.6%
No	7362	93.6%	7313	93.0%	7266	92.4%
	Mean (SD)					
CES-D-11	3.20 (4.38)		3.33 (4.51)		4.04 (4.91)	
Socioeconomic Deprivation	1.33 (1.24)		1.29 (1.21)		1.25 (1.19)	
Family Relationship Satisfaction	5.47 (0.92)		5.57 (0.90)		5.54 (0.94)	
Self-esteem	30.64 (3.79)		31.12 (3.90)		30.73 (4.08)	

Note. Values are presented as n (%) or mean (SD), as appropriate. For socioeconomic deprivation, the reported mean represents the average of the summed deprivation scores (range: 0–21) across participants.

**Table 4:** Means, standard deviations, and correlations between variables.

2017 year	**1**	**2**	**3**	**4**	**5**	**6**	**7**
1. Depression	1						
2. Age	0.254 ***	1					
3. Gender	0.167 ***	0.055 ***	1				
4. Self-esteem	−0.526 ***	−0.339 ***	−0.112 ***	1			
5. Socioeconomic deprivation	0.289 ***	0.157 ***	0.009	−0.282 ***	1		
6. Family relationship satisfaction	−0.407 ***	−0.196 ***	−0.063 ***	0.465 ***	−0.251 ***	1	
7. Basic Livelihood Security System	0.216 ***	0.100 ***	0.068 ***	−0.233 ***	0.289 ***	−0.216 ***	1
2019 year	**1**	**2**	**3**	**4**	**5**	**6**	**7**
1. Depression	1						
2. Age	0.291 ***	1					
3. Gender	0.154 ***	0.055 ***	1				
4. Self-esteem	−0.483 ***	−0.335 ***	−0.098 ***	1			
5. Socioeconomic deprivation	0.260 ***	0.151 ***	−0.001	−0.220 ***	1		
6. Family relationship satisfaction	−0.340 ***	−0.162 ***	−0.058 ***	0.446 ***	−0.182 ***	1	
7. Basic Livelihood Security System	0.234 ***	0.117 ***	0.064 ***	−0.238 ***	0.305 ***	−0.215 ***	1
2021 year	**1**	**2**	**3**	**4**	**5**	**6**	**7**
1. Depression	1						
2. Age	0.326 ***	1					
3. Gender	0.161 ***	0.055 ***	1				
4. Self-esteem	−0.583 ***	−0.363 ***	−0.077 ***	1			
5. Socioeconomic deprivation	0.273 ***	0.168 ***	0.009	−0.243 ***	1		
6. Family relationship satisfaction	−0.353 ***	−0.162 ***	−0.056 ***	0.435 ***	−0.181 ***	1	
7. Basic Livelihood Security System	0.248 ***	0.125 ***	0.063 ***	−0.265 ***	0.288 ***	−0.211 ***	1

N = 7867, *** *p* < 0.001.

**Table 5:** Generalized Estimating Equation Model for Socioeconomic Deprivation and Family Relationship Satisfaction.

Variables	Depression			
	B	95% CI	Wald χ^2^	*p*-Value
Age	0.035	[0.031, 0.039]	296.623	0.000
Male	−1.019	[−1.128, −0.910]	336.124	0.000
Female	Ref			
Self-esteem	−0.457	[−0.478, −0.436]	1858.156	0.000
Socioeconomic deprivation	0.485	[0.432, 0.539]	317.613	0.000
Family relationship satisfaction	−0.685	[−0.764, −0.607]	292.489	0.000

**Table 6:** Generalized Estimating Equation Model Including the Interaction Between Socioeconomic Deprivation and Family Relationship Satisfaction.

Variables	Depression			
	B	95% CI	Wald χ^2^	*p*-Value
Age	0.036	[0.032, 0.040]	313.536	0.000
Male	−1.016	[−1.125, −0.908]	336.052	0.000
Female	Ref			
Self-esteem	−0.455	[−0.476, −0.435]	1828.283	0.000
Socioeconomic deprivation	1.383	[1.075, 1.691]	77.509	0.000
Family relationship satisfaction	−0.406	[−0.512, −0.299]	55.664	0.000
Socioeconomic deprivation × Family relationship satisfaction	−0.174	[−0.229, −0.118]	37.662	0.000

**Table 7:** Generalized estimating equation of Basic Livelihood Security System.

Variables	Depression			
	B	95% CI	Wald χ^2^	*p*-Value
Age	0.035	[0.031, 0.039]	292.598	0.000
Male	−1.012	[−1.122, −0.902]	325.695	0.000
Female	Ref			
Self-esteem	−0.510	[−0.530, −0.490]	2463.099	0.000
Socioeconomic deprivation	0.471	[0.416, 0.525]	287.102	0.000
Basic Livelihood Security System	1.281	[0.963, 1.599]	62.356	0.000

**Table 8:** Interaction term between Socioeconomic deprivation and Basic Livelihood Security System.

Variables	Depression			
	B	95% CI	Wald χ^2^	*p*-Value
Age	0.035	[0.031, 0.039]	297.322	0.000
Male	−1.012	[−1.122, −0.902]	325.936	0.000
Female	Ref			
Self-esteem	−0.510	[−0.530, −0.489]	2458.062	0.000
Socioeconomic deprivation	0.450	[0.393, 0.507]	237.018	0.000
Basic Livelihood Security System	0.961	[0.482, 1.441]	15.455	0.000
Socioeconomic deprivation × Basic Livelihood Security System	0.135	[−0.032, 0.302]	2.506	0.113

## Data Availability

The datasets analyzed during the current study are publicly available from the Korea Welfare Panel Study (KoWePS) at http://www.kihasa.re.kr (accessed on 14 March 2023).

## Supplementary Materials

The following supporting information can be downloaded at: https://www.mdpi.com/article/10.3390/bs16091463/s1, Table S1. Items and Scoring of the Socioeconomic Deprivation Measure.
