# Supplementary material for: Socioeconomic Deprivation’s Effect on Depression: Family Satisfaction’s Moderating Role
Source: Behav Sci (Basel). 2026 Aug 24;16(9):1463. doi: 10.3390/bs16091463 (PMC13603880; doi:10.3390/bs16091463)
Supplement: Supplementary file 1 [file behavsci-16-01463-s001.zip › behavsci-4471194-supplementary.pdf]

Table S1. Items and Scoring of the Socioeconomic Deprivation Measure

**Domain**	No	Item
Nutrition	1	Ran out of food but could not afford to buy more because of financial difficulties
	2	Unable to afford balanced meals because of financial difficulties
	3	Adults in the household reduced meal size or skipped meals because there was not enough money for food
	4	Ate less than the amount considered necessary because there was not enough money for food
	5	Was hungry but could not eat because there was not enough money for food
Housing	6	Moved because rent was overdue for two months or more or could not be paid
	7	Unable to heat the home during winter because of financial difficulties
	8	Housing did not meet standards for structural durability and appropriate heat-, fire-, moisture-, and insulation-resistant materials
	9	Housing did not have adequate soundproofing, ventilation, lighting, or heating facilities
	10	Housing environment was unsuitable for living because of noise, vibration, odor, or air pollution
	11	Housing was not safe from natural hazards such as tidal waves, floods, landslides, or cliff collapse
	12	Electricity, telephone, or water service was disconnected because bills could not be paid
Social security	13	Not contributing to the National Pension
	14	Not enrolled in National Health Insurance or experienced nonpayment of premiums
Occupational and economic	15	Unemployed
	16	Classified as a low-income household based on equivalized household income
	17	Experienced working in a hazardous environment
Health and medical	18	Respondent or family member was unable to visit a hospital because of financial difficulties
	19	Presence of a chronic disease
Social deprivation	20	Unable to pay utility bills by the due date
	21	Presence of a household member with credit delinquency

[1] **Note**. Each item was dichotomously coded as 0 = no deprivation and 1 = deprivation. Items were summed without differential weighting, yielding a possible total score ranging from 0 to 21.
